# Nasal epithelial cells to assess in vitro immune responses to respiratory virus infection in pregnant women with asthma

**DOI:** 10.1186/s12931-019-1225-5

**Published:** 2019-11-20

**Authors:** Rebecca L. Vanders, Alan Hsu, Peter G. Gibson, Vanessa E. Murphy, Peter A. B. Wark

**Affiliations:** 1 Priority Research Centre for Healthy Lungs, The University of Newcastle, Newcastle, NSW Australia; 2 Viruses, Infection & Immunity, Vaccines & Asthma (VIVA), Hunter Medical Research Institute, Newcastle, NSW Australia; 3 The Department of Respiratory and Sleep Medicine, John Hunter Hospital, Newcastle, NSW Australia; 4 Priority Research Centre GrowUpWell™, The University of Newcastle, Callaghan, NSW 2305 Australia

**Keywords:** Pregnancy, Asthma, Nasal cells, Respiratory infections, Influenza, Rhinovirus, In vitro cultures

## Abstract

Respiratory virus-induced asthma exacerbations occur frequently during pregnancy and are associated with adverse outcomes for mother and child. Primary nasal epithelial cells (pNECs) provide a useful method to study immune responses in pregnancy. pNECs were obtained by nasal brushings from pregnant and non-pregnant women with and without asthma. pNECS were infected in vitro with major group Rhinovirus 43 (RV43) and seasonal influenza (H3N2). Following infection, pNECs showed measurable quantities of interferon (IFN)-λ, IL-1β, IL-8, IP-10 and MIP1-α. pNECs provide a safe and effective method for studying respiratory epithelial cell responses during pregnancy.

## Introduction

Asthma is a common comorbidity during pregnancy and asthma exacerbations during pregnancy are frequently caused by respiratory virus infections, such as rhinovirus and influenza virus [1]. This was observed in a prospective study where 71% of pregnant asthmatic women experienced a probable cold compared to 46% of pregnant non-asthmatic women. These women also experienced multiple colds and showed greater symptom severity compared to pregnant non-asthmatics [1].

The influenza virus frequently cause severe illness in ‘high risk groups’ including asthmatics and pregnant women. For example in one study, which included 6,277,508 hospitalised pregnant women over four influenza seasons [2], it was observed that for every 1000 pregnant women hospitalised, 3.4 [95% CI 3.3–3.6] had a respiratory illness.

Bronchial epithelial cells and blood cells isolated from asthmatics display attenuated antiviral and heightened inflammatory immune responses following in vitro infection with respiratory viruses, which positively correlate with worsened clinical symptoms in these patients [3, 4]. We have also identified that peripheral blood mononuclear cells (PBMCs) isolated from pregnant women with and without asthma, and stimulated in vitro with rhinoviruses or influenza virus (H1N1pdm09) exhibit attenuated antiviral immunity [5–7].

Despite the obvious benefits of primary bronchial epithelial cell (pBEC) cultures in exploring respiratory epithelial cell responses from subjects with respiratory diseases, using bronchoscopies to collect pBECs can be expensive, requires special training to perform, is invasive, and most importantly, is not safe to perform during pregnancy [8].

The ability to obtain epithelial cells from the respiratory tract using a method of collection suitable during pregnancy, would be of great value. Nasal brushings provide one alternative method that has gained popularity for obtaining respiratory epithelial cells because it is inexpensive, quick and easy to perform and far less invasive [9]. In this study, we describe for the first time, an effective and safe method for obtaining and culturing primary nasal epithelial cells (pNECs) specifically from pregnant women with and without asthma. We show that the cells obtained by this method have similar morphological and growth characteristics to pBECs. These pNECs can be effectively cultured as undifferentiated monolayers and infected in vitro with respiratory viruses to elicit both antiviral and inflammatory immune responses.

## Methods

### Subjects

pNECs were collected from *n* = 6 pregnant asthmatics, n = 6 pregnant non-asthmatics, *n* = 3 non-pregnant asthmatics and n = 6 non-pregnant controls. Pregnant women were participating in a randomised controlled trial at the John Hunter Hospital, and were recruited from the antenatal clinic as described previously [10]. Non-pregnant women were recruited from the Hunter Medical Research Institute Register and from John Hunter Hospital respiratory staff as described previously [5]. Inclusion criteria were females aged 18–40 years old and for pregnant women, over 18 weeks gestation. For asthmatics, doctor’s diagnosis was also required. Women were excluded if they had another chronic medical illness, cold/flu symptoms (assessed using Common Cold Questionnaire [11]) within the month prior to sample collection, and for asthmatics, any loss of asthma control within the past month (Asthma Control Questionnaire and asthma control criteria from the GINA guidelines [12]). Ethics approval was obtained from the Hunter New England Human Research Ethics Committee and the University of Newcastle Research Ethics Committee. Written informed consent was obtained from all participants prior to subject characterisation and sample collections.

### Viruses

RV43, a major group rhinovirus, was a clinical isolate obtained in 2005 from sputum and nasal/throat swabs collected from subjects over 7 years of age presenting to John Hunter Hospital Emergency Department with an acute asthma exacerbation. RV43 was propagated in ICAM-1 expressing rhabdomyosarcoma cells (RD-ICAMs; ATCC, Manassas, VA, USA) in T175 tissue culture flasks, at 37 °C/5% CO_2_ in Dulbeco’s modified eagles medium (DMEM Sigma-Aldrich Co, Castle Hill, NSW, Australia) with 5% fetal bovine serum (FBS; SAFC Biosciences, Lenexa, Kansas, USA). For subsequent pNEC infections, we used an MOI of 20. Whilst this is a high MOI, we found that this was the most effective viral concentration to induce a maximal immune response from nasal cells with minimal cell death. Human H3N2 (A/Wellington/43/2006) was obtained from the World Health Organisation (WHO) in 2006. H3N2 was propagated in Madin-Darby Canine Kidney Cells (MDCKs; ATCC, Manassas, VA, USA) in DMEM with 5% FBS. For subsequent pNEC infections we used an MOI5 (4 × 10^5^ pful/ml).

### Nasal brushings

To obtain pNECs, a small sterile interdental brush (Curaden Swiss, Australia) was inserted into the anterior region of the inferior meatus and brushed back and forth, then up and down several times. The brush was then dipped into sterile Dulbeco’s PBS (Gibco Invitrogen, Australia Pty Ltd. Vic Australia) in 1.5 ml microcentrifuge tubes and vigorously rubbed up and down to loosen the attached cells. This procedure was repeated twice in each nostril for a total of four brushings.

### pNEC undifferentiated submerged monocultures

The microcentrifuge tubes were centrifuged at 300×g for 5 min, supernatant was removed and each cell pellet resuspended in 250 μl of complete bronchial epithelial growth media (BEGMc; LONZA Clonetics Walkersville USA). Cell suspensions were pooled and transferred to a T25 tissue culture flask, pre-coated with placental collagen (0.8 μg/cm^2^ Sigma-Aldrich, Castle Hill Australia) and made up with 3 ml of BEGMc with 10% fetal bovine serum (FBS). After 24 h of culture at 37 °C (to allow cell attachment), the media was replaced with 3.5-5 ml of fresh BEGMc without FBS. Cultures were than maintained in BEGMc, with media changes every 2–3 days, until cells reach 80% confluence; usually 3–4 weeks. At confluence, pNECs were subcultured by adding trypsin (Gibco, 10x trypsin Sigma-Aldrich, Castle Hill Australia), incubated at 37 °C for 5-10 min (or until nearly all cells were floating as visualised by microscopy) and then inactivated using FBS. The cell suspension was centrifuged at 550×g for 5 min, and the resultant pellet was resuspended in BEGMc. pNECs were then seeded into 24 well plates (pre-coated with collagen 0.8 μg/cm2) at a final concentration of 10^4^ cells in 500 μl BEGMc. Media was replaced every second day until cells reach 60–80% confluence (usually 1-2 weeks).

### pNEC air-liquid Interface (ALI) differentiated cultures

pNECs from three pregnant women were successfully cultured as ALI cultures (see online supplement). Due to limited patient samples, all subsequent respiratory virus infections in pregnant and non-pregnant women, with and without asthma were performed in submerged cultures.

### pNEC in vitro infection with respiratory virus

Semi-confluent cells (~ 80%) were first washed in PBS, followed by the addition of RV43 (MOI20) or H3N2 (MOI5 or 4x10^5^pfu/ml) in a final volume of 500 μl bronchial epithelial cell basal media (BEBM; LONZA Clonetics Walkersville USA) with 0.1% insulin-transferrin-sodium selenite liquid media supplement (ITS+ 1 Sigma-Aldrich Co, Castle Hill, NSW, Australia). During infection, pNECs were incubated either at room temperature with shaking (RV43) or 37 °C without shaking (H3N2). After one hour, virus-containing media was removed, cells were washed twice with PBS and 1 ml of fresh BEBM with 0.1% ITS was added to each well. All experiments were performed in duplicate, or where possible, triplicate. After 48 h of incubation at 33 °C (RV43) or 35 °C (H3N2), supernatant was collected, briefly centrifuged to remove any cellular debris and stored at -80 °C for subsequent cytokine/chemokine and viral analyses.

### Protein measurements

IFN-λ was measured from culture supernatant using R&D Duoset (Minneapolis, Canada) according to manufacturer’s instructions. IL1-β, IL-8, IP-10 and MIP1- α were measured using cytometric bead array from BD Bioscience (CA, USA) according to manufacturer’s instructions.

### Cell viability

To determine cell viability, cells were analysed by flow cytometry using the PE Annexin V Apoptosis Kit I (BD Bioscience, CA, USA), according to manufacturer’s instructions.

### Statistics

Data were analysed using Graph Pad Prism 8 (La Jolla, CA, USA). Any outliers were first removed using the ROUT outlier test with Q = 1%. Normality of the data was then tested using the D’Agostino-Pearson omnibus normality test or when numbers were too small, using the Shapiro-Walk normality test. One-way ANOVA (or rank-sum) with corrected multiple comparisons between all the groups was then performed.

## Results

### pNECs can be obtained from pregnant women by nasal brushings and cultured to produce monolayers

**Fig. 1:**
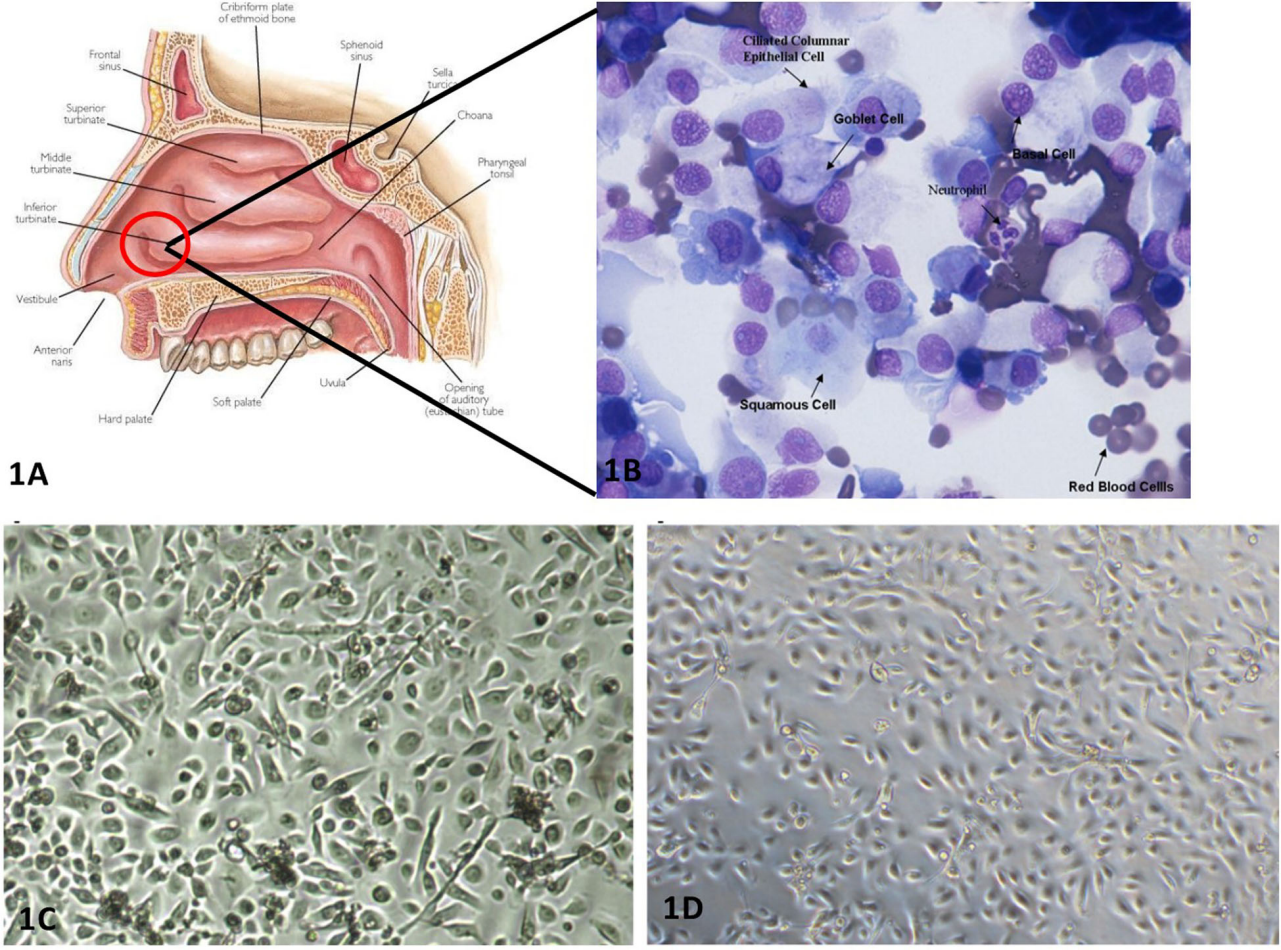
pNECs obtained from nasal brushings are true representatives of epithelial cells in the upper respiratory tract. (A) Primary nasal epithelial cells were obtained by nasal brushing from the anterior region of the inferior nasal turbinate. (B) Cellular content from the brushings was anlysed directly by cytospins, prepared using 100 μl aliquots of cell suspensions, stained with MGG. (C) pNECs obtained by nasal brushings were cultured in tissue culture flasks until confluent and produced the same “cobblestone-like” appearance as (D) pBECs

pNECs were obtained by brushing the anterior region of the inferior turbinate (Fig. 1a). Mixtures of cell types are obtained following the brushings; including ciliated and non-ciliated columnar epithelial cells, granulocytes, squamous epithelial cells and red blood cells (Fig. 1b). Once transferred to collagen-coated tissue culture flasks, specifically supplemented with media containing FBS, pNECs will adhere to the flasks overnight, whilst all other cell types will remain in suspension and can be removed by rinsing with PBS. After several weeks of culture, confluent monolayers are observed and display the typical ‘cobble-stone’ morphology (Fig. 1c) also observed with cultured pBECs (Fig. 1d).

### Infection of pNECs with respiratory viruses induces a host immune response

**Fig. 2:**
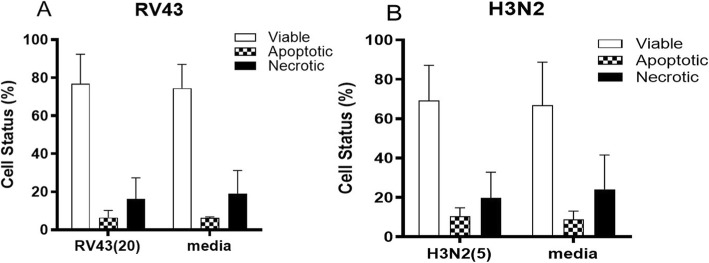
Infection of pNEC cultures induces minimal cell death. Submerged cultures of pNECs from pregnant and non-pregnant women with and without asthma were infected with (A) RV43 or (B) H3N2. Infection resulted in cytopathic effects evidenced as small holes in the monolayer, however cell viability was still between 70 and 80% with both RV43 and H3N2. Data represented as mean +/− SEM

Submerged cultures of pNECs were infected with the major group rhinovirus RV43 and with a seasonal influenza strain, H3N2 (Fig. 2). Infection with respiratory virus resulted in the typical cytopathic effect (CPE) evidenced as small “holes” in the confluent monolayer which is otherwise absent in cells cultured with media alone. Viral infection with both respiratory viruses, resulted in minimal cellular apoptosis or necrosis; with 70–80% of cells being viable following infection (Fig. 2a and b).

### Infection of pNECs with respiratory viruses induces antiviral and inflammatory cytokines and chemokines

**Fig. 3:**
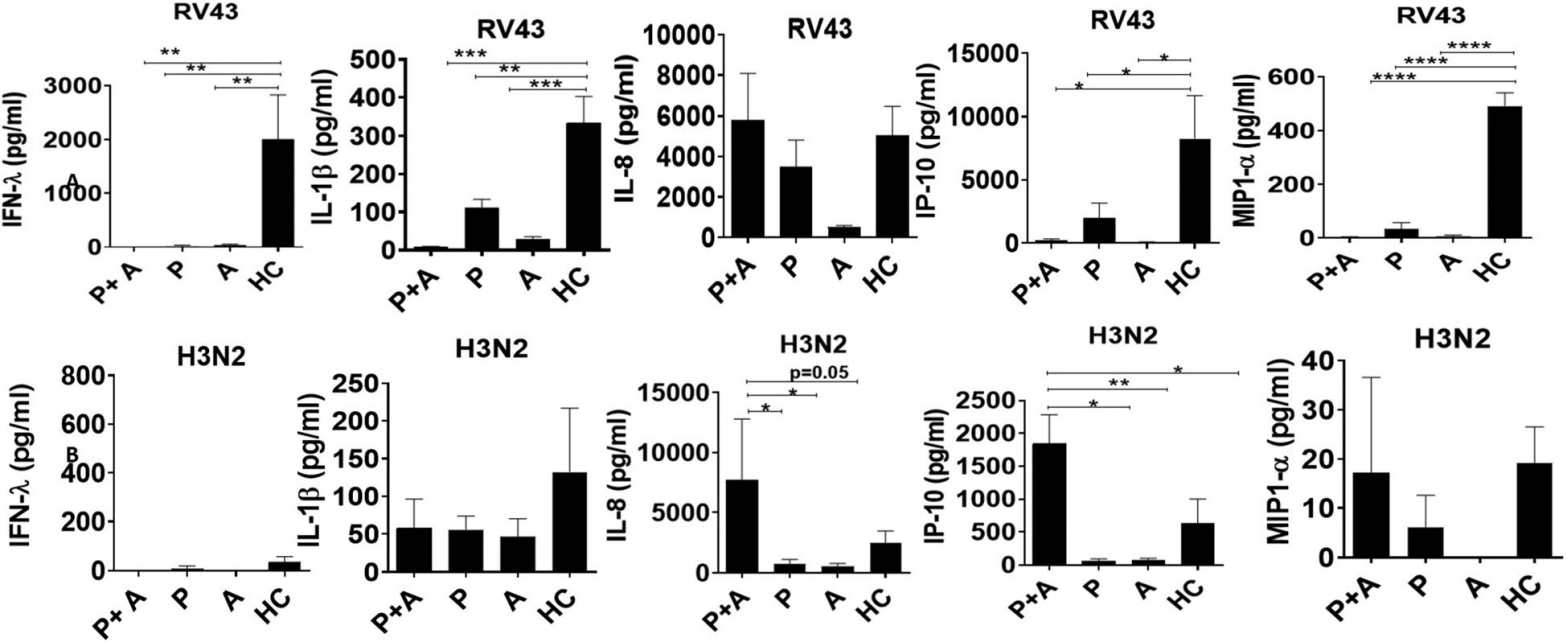
Infection of pNEC cultures induces an array of antiviral and inflammatory cytokines and chemokines from all infected groups. Following infection of submerged cultures from pregnant (P), pregnant asthmatic (P + A), non-pregnant asthmatic (A) and non-pregnant non-asthmatic women (i.e. healthy controls; HC) resulted in the production of IFN-λ, IL-1β, IL-8, IP-10 and MIP1-α. *p < 0.05, **p < 0.01, ****p < 0.00001. Data represented as mean +/− SEM

Following infection with RV43, all groups produced measurable levels of IFN-λ, IL1-β, IL-8, IP-10 and MIP1- α (Fig. 3a). RV43 infection resulted in a signifcant decrease in IFN-λ, IL1-β, IP-10 and MIP-1α production in pNECs from pregnant and asthmatic groups compared to the healthy controls.

Similarly, following infection with H3N2, all groups produced measurable levels of IFN-λ, IL1-β, IL-8, IP-10 and MIP1- α (Fig. 3b). A signficant increase was observed in IL-8 and IP-10 from pregnant and asthmatic groups compared to healthy controls.

## Discussion

During pregnancy, asthmatic women show increased susceptibility and disease severity to respiratory virus infections, and are known to have reduced antiviral responses in peripheral blood cells [5, 6]. However no knowledge of the respiratory epithelial role has yet been established, largely due to inability to collect bronchial cells during pregnancy. Since the nasal epithelium is the primary portal for viral entry and immediate replication, and acts not only as a physical barrier, but actively prevents airway damage by inducing its own unique immune responses [13], studying the responses of these nasal cells during pregnancy would be useful.

In this study, we show for the first time that pNECs from pregnant women with and without asthma can be obtained by nasal brushings, cultured and infected with respiratory viruses, and show measurable levels of both inflammatory and antiviral cytokines and chemokines.

We show that IFN-λ, the primary IFN released from nasal epithelial cells, responsible for stopping viral spread to lower airways [14], is significantly reduced following rhinovirus infection of pNECs from pregnant women with and without asthma, as well as non-pregnant women with asthma, compared to healthy controls. In line with this, MIP1α, a chemotactic cytokine that recruits B cells and T cells to the site of infection [15], was also significantly reduced in these groups compared to healthy controls. Collectively these findings indicate a suppression in the host cell antiviral response to rhinovirus infection in pregnancy and asthma, providing a plausible explanation for the increased susceptibly observed in these women following infection.

We also found that IP-10, a biomarker that positively correlates with acute respiratory infection (ARI) following influenza virus infection [16], was significantly increased in pregnant women with asthma compared to all controls. Since nasal cells are the first respiratory cells to be infected with the influenza virus, these findings highlight the severity of the influenza virus infection for pregnant women, especially those with underlying respiratory conditions like asthma. Following RV43 infection, we saw the opposite response for IP-10 production in pregnant asthmatic women, with a significant decrease compared to healthy controls. IP-10 production from bronchial epithelial cells has been shown to correlate with asthma severity following rhinovirus infections [15] however a recent study using nasal cells from healthy showed increased IP-10 production following rhinovirus infection [17]; indicating that decreased IP-10 production in nasal cells may lead to increased risk for viral infection and spread to the lower airways.

In 2013 it was shown for the first time that rhinovirus infection induces inflammasome activation in bronchial epithelial cells [18], and our findings of altered IL1-β activity in nasal cells, particularly following RV43 infection, highlight that viral inflammasomes may also play a role in nasal epithelium responses in pregnancy and asthma.

Collectively, these findings highlight the importance of developing suitable in vitro models of respiratory virus infections in nasal epithelial cultures from pregnant women with and without asthma. We acknowledge that larger sample sizes and ALI cultures will need to be utilized in the future to provide a more clinically accurate representation of the nasal respiratory response in pregnant women following respiratory virus infections. This current study therefore, provides a suitable platform for future studies, which will help us understand the changes taking place in maternal respiratory cells following respiratory virus infections and the ability to develop suitable therapies targeting the nasal epithelium.

## Data Availability

Not applicable.
